# Supplementary material for: Metachromatic Leukodystrophy in Morocco: Identification of Causative Variants by Next-Generation Sequencing (NGS)
Source: Genes (Basel). 2024 Nov 26;15(12):1515. doi: 10.3390/genes15121515 (PMC11675868; doi:10.3390/genes15121515)
Supplement: Supplementary file 1 [file genes-15-01515-s001.zip › genes-3302088-supplementary.pdf]

Table S1. List of the 101 genes anayzed by the STP v3 panel.

STP Panel v 3

Gene #	gene symbol
1	*A4GALT*
2	*ABCA1*
3	*ABHD5*
4	*ACER1*
5	*AGA*
6	*AK1*
7	*ANK1*
8	*APOE*
9	*AP3B1*
10	*ARSA*
11	*ARSB*
12	*ASAH1*
13	*ASAH2*
14	*BIN1*
15	*CHIT1*
16	*CLCN7*
17	*CLN3*
18	*CLN5*
19	*CLN6*
20	*CLN8*
21	*CTNS*
22	*CTSA*
23	*CTSK*
24	*EPB41*
25	*EPB42*
26	*FUCA1*
27	*G6PD*
28	*GAA*
29	*GALC*
30	*GALNS*
31	*GATA1*
32	*GBA*
33	*GBA2*
34	*GBA3*
35	*GLA*
36	*GLB1*
37	*GM2A*
38	*GNPTAB*
39	*GNPTG*
40	*GNS*
41	*GPI*
42	*GRN*
43	*GUSB*
44	*HEXA*
45	*HEXB*
46	*HGSNAT*
47	*HYAL1*
48	*IDS*
49	*IDUA*
50	*ITCH*
51	*KIT*
52	*LAMP1*
53	*LAMP2*
54	*LAMP3*
55	*LIPA*
56	*LRP2*
57	*LRRK2*
58	*LYST*
59	*MAN2B1*
60	*MANBA*
61	*MCOLN1*
62	*MRC1*
63	*MTX1*
64	*NAGA*
65	*NAGLU*
66	*NEU1*
67	*NPC1*
68	*NPC2*
69	*NPR2*
70	*NT5C3A*
71	*PARK7 (DJ1)*
72	*PIEZO1*
73	*PINK1*
74	*PKLR*
75	*PPT1*
76	*PRF1*
77	*PRKN (PARK2)*
78	*PSAP*
79	*RAB27A*
80	*SCARB2*
81	*SGSH*
82	*SLC17A5*
83	*SLC4A1*
84	*SMPD1*
85	*SNCA*
86	*SNX10*
87	*SORT1*
88	*SPTA1*
89	*SPTB*
90	*STX11*
91	*STXBP2*
92	*SUMF1*
93	*TCIRG1*
94	*TFEB*
95	*TMEM175*
96	*TNFSF11*
97	*TPP1*
98	*UGCG*
99	*UNC13D*
100	*VDR*
101	*VPS35*

Lysosomal storage disorders	49
Erythrocyte membrane disorders	6
Hemolytic anemia	6
Other Hematological Disorders	8
Other disorders	8
Involved in lysosomal function	17
Biomarkers	1
Parkinson disease	6
**Total**	**101**

Table S2. Genotype-phenotype summary

Patient	Homozygous variants		Clinical features	MRI findings	Arylsulfatase A activity*
	DNA	Protein			
PET170	c.854+1dup	p.(Pro286Thrfs*2)	Psychomotor regression at 18 months old<br>Hypotonia<br>Peripheral neuropathy<br>Ataxia	T2 and FLAIR hypersignals in white matter<br>Hypoplasia of corpus callosum<br>Ventricles and cerebellar abnormalities	15.21
PET173	c.640G>A	p.(Ala214Thr)	Psychomotor regression at 30 months old	T2 and FLAIR hypersignals in periventricular white matter and semioval center<br>T2 hypersignals affecting corpus callosum<br>Normal ventricles and cerebellum	26.25
PET174	c.640G>A	p.(Ala214Thr)	Psychomotor regression at 36 months old	FLAIR hypersignals in periventricular white matter	15.42

* Normal reference values: 35.16-177.12 (units: nmol/mL/4 hours)

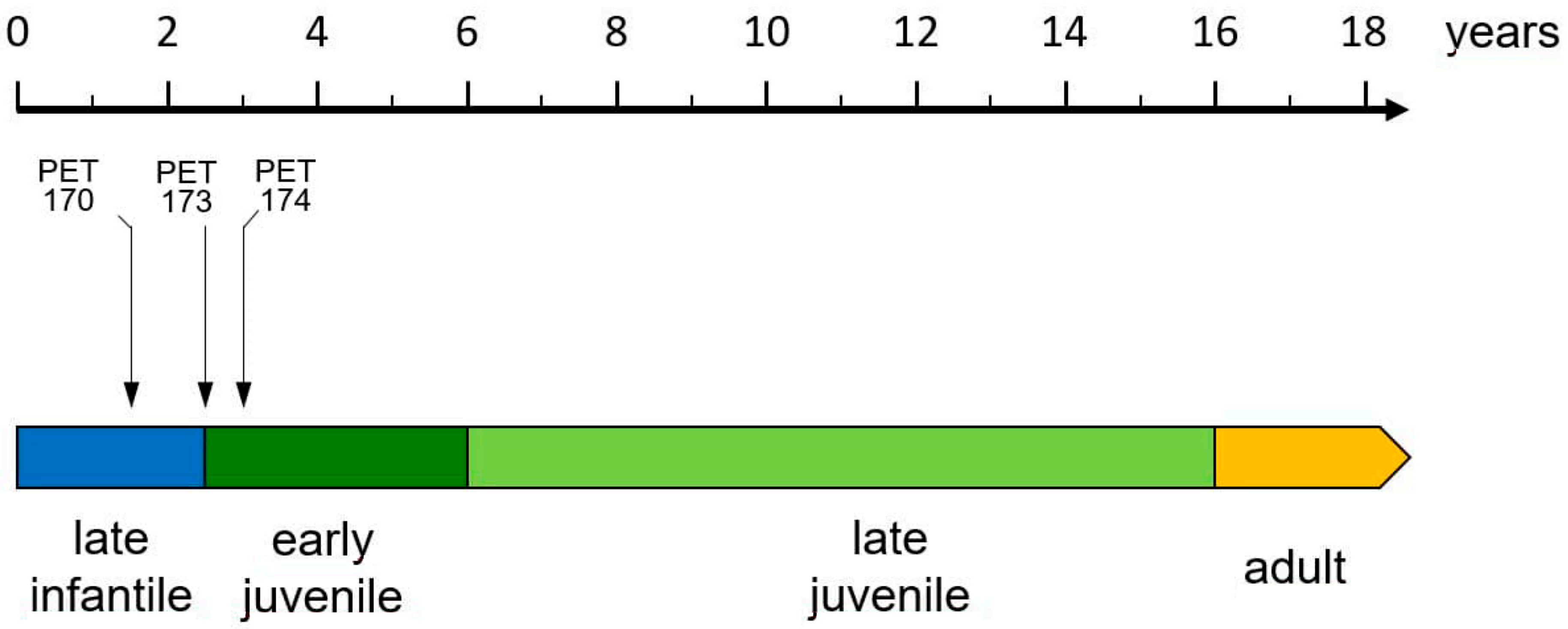

Figure S1. Timeline of onset of psychomotor regression in the three patients described in this work, PET170 (homozygous for c.854+1dup, with earliest onset) from family 1 and PET173 and PET174 (both homozygotes for c.640G>A) from family 2.
